# The emerging role of neutrophils in thrombosis—the journey of TF through NETs

**DOI:** 10.3389/fimmu.2012.00385

**Published:** 2012-12-18

**Authors:** Konstantinos Kambas, Ioannis Mitroulis, Konstantinos Ritis

**Affiliations:** First Department of Internal Medicine, University General Hospital of Alexandroupolis, Democritus University of ThraceAlexandroupolis, Greece

**Keywords:** neutrophil extracellular traps, thrombosis, tissue factor, neutrophil, coagulation cascade

## Abstract

The production of TF by neutrophils and their contribution in thrombosis was until recently a matter of scientific debate. Experimental data suggested the *de novo* TF production by neutrophils under inflammatory stimuli, while others proposed that these cells acquired microparticle-derived TF. Recent experimental evidence revealed the critical role of neutrophils in thrombotic events. Neutrophil derived TF has been implicated in this process in several human and animal models. Additionally, neutrophil extracellular trap (NET) release has emerged as a major contributor in neutrophil-driven thrombogenicity in disease models including sepsis, deep venous thrombosis, and malignancy. It is suggested that NETs provide the scaffold for fibrin deposition and platelet entrapment and subsequent activation. The recently reported autophagy-dependent extracellular delivery of TF in NETs further supports the involvement of neutrophils in thrombosis. Herein, we seek to review novel data regarding the role of neutrophils in thrombosis, emphasizing the implication of TF and NETs.

## Historical introduction

The role of neutrophils in the interface between inflammation and thrombosis remains a controversial scientific issue. Even though there is significant evidence that suggests a critical role for neutrophils in the thrombotic process (Lerner et al., 1971), their impact on thrombogenesis was until recently questioned. The ability of neutrophils to enhance or trigger *in vivo* thrombotic events via production and expression tissue factor (TF), the major *in vivo* initiator of coagulation (Rapaport and Rao, 1995; Bächli, 2000), is a matter of debate. During the past decade, experimental evidence using not physiologic inflammatory conditions reinforced the view that neutrophils acquire rather than produce TF (Osterud et al., 2000; Egorina et al., 2008). As a result, the significance of this cell population in thrombosis was considered minor and the study of neutrophils was not included in research models of thrombosis.

During the last few years several studies elucidated the crucial role of neutrophils in thrombosis (Looney et al., 2006; Zernecke et al., 2008; Darbousset et al., 2012; von Brühl et al., 2012). Neutrophil depletion was shown to be able to reverse *in vivo* experimental thrombosis, thus supporting the significance of this population (von Brühl et al., 2012). Several studies support the *in vivo* and *ex vivo* TF production by neutrophils (Maugeri et al., 2006; Ritis et al., 2006; Redecha et al., 2007, 2008; Kambas et al., 2008; Rafail et al., 2008; Kourtzelis et al., 2010).

The description of neutrophil extracellular trap (NET) release by neutrophils revealed a novel inflammatory role of these cells (Brinkmann et al., 2004; Clark et al., 2007; Fuchs et al., 2007; Medina, 2009; Mitroulis et al., 2011). Moreover, recent experimental data regarding the bridging of autophagy with neutrophils and immunity (Mitroulis et al., 2010), added a novel view on their functions. The implication of autophagy in NET release and TF delivery to NETs and the linkage between NETs and thrombosis suggest a critical role for neutrophils in the interaction between inflammatory and thrombotic pathways. Moreover, the attenuation of thrombotic manifestations in thrombotic animal models by neutrophil depletion demonstrates the contribution of these cells in thrombosis (von Brühl et al., 2012). The expression of produced and/or acquired TF by neutrophils is an attractive and realistic scenario for the pathogenesis of thrombotic events that characterize several inflammatory disorders, including sepsis (Aras et al., 2004), ANCA-associated vasculitis and Behçet's disease (Tomasson et al., 2009), or inflammatory bowel disease (Miehsler et al., 2004).

## TF: the orchestrator of coagulation

TF is a 47 kDa transmembrane glycoprotein that shares high homology in secondary and tertiary structure with interferon γ receptors and is a member of the human class II cytokine receptor family (Bazan, 1990). Currently, TF is considered as the main *in vivo* initiator of coagulation (Rapaport and Rao, 1995; Bächli, 2000; Manly et al., 2011). The presence of a multitude of binding sites in the gene's promoter region indicates multi-potency of expression in a large variety of cells and under a vast array of stimuli. Under normal conditions TF is not expressed in endothelial cells (Wilcox et al., 1989), but only in sub-endothelial tissue, thus creating a protecting envelope between blood and sites of expression (Drake et al., 1989; Fleck et al., 1990). However, under specific inflammatory conditions TF is expressed in endothelial cells and myeloid leukocytes (Parry and Mackman, 1995; Armesilla et al., 1999; Maugeri et al., 2006; Ritis et al., 2006; Kambas et al., 2008; Rafail et al., 2008; Kourtzelis et al., 2010).

There is emerging evidence indicating the presence of circulating TF in blood (blood-borne TF). There are three potential sources of blood-borne TF—peripheral blood cells (Drake et al., 1989; Ritis et al., 2006; Kambas et al., 2008; Kourtzelis et al., 2010), microparticles (MPs) (Mallat et al., 2000) and the soluble alternative spliced variant of TF (Bogdanov et al., 2003). Although monocytes have been reported to constitutively express TF and while this cell population is considered the main source of TF-bearing MPs (Aleman et al., 2011), there are emerging evidence indicating the possible implication of other cell populations in the generation of blood-borne TF.

Intraluminal exposure of TF located on a serine-rich phospholipid membrane activates FVII, forming TF/FVIIa complex (Bach, 1988). This complex is able to activate FX, which in turn results in thrombin, which is responsible for thrombus stabilization (Monroe et al., 2002). Moreover, membrane-embedded TF is usually in inactive coagulant state (cryptic) while it requires activation to reach its full potency (decryption) (Rao et al., 2012). However, the mechanism behind the activation of circulating TF is not yet elucidated and such information would provide a significant breakthrough in the understanding of *in vivo* thrombosis.

Nevertheless, apart from the role of the extrinsic coagulation system (TF—thrombin axis) in thrombosis, this system has been implicated in several non-thrombotic models such as angiogenesis, tumor growth and metastasis, inflammation, and fibrosis. The serine proteases of this pathway, namely TF/VIIa, Xa, and thrombin, are able to signal through the protease activated receptor (PAR) receptor family to produce intracellular signals (Coughlin, 2005) *via* phosphoinositide 3-kinase (PI3K), Src tyrosine kinase, extracellular signal-regulated kinase (ERK), and mitogen-activated protein kinase (MAPK) pathways (Ramachandran and Hollenberg, 2008). The activation of these pathways results in the secretion of cytokines and chemokines implicated in several biological functions (Coughlin, 2005).

## Inflammation and thrombosis: a reciprocal process

Increased prevalence of venous thrombotic events is a long standing observation in patients suffering from infectious and sterile inflammatory disorders. Venous thrombosis constitutes a major morbidity and mortality factor in inflammatory diseases, including sepsis, systemic lupus erythematosus (SLE), inflammatory bowel disease, or vasculitis (Zöller et al., 2012). Additionally, recent clinical data derived from patients with rheumatoid arthritis and SLE support the critical role of inflammation in accelerated atherothrombosis (Santos et al., 2012). Experimental evidence links the observed thrombogenicity with TF-dependent activation of extrinsic coagulation cascade. Increased TF expression by endothelial and blood cells exposed to inflammatory mediators is proposed as an essential part of the pathogenic mechanism for arterial and venous thromboembolism that characterizes inflammatory disorders (Mackman, 2009). These observations indicate a potential triggering role of inflammation in thrombosis. However, the relationship between inflammation and thrombosis is bidirectional since thrombosis can reignite inflammation creating a persistent or recurrent inflammatory environment. TF-thrombin axis enhances the inflammatory response in several clinical models such as arthritis (Busso et al., 2003) antiphospholipid syndrome (APS) (Ritis et al., 2006; Redecha et al., 2007, 2008), ischemia/reperfusion injury (Loubele et al., 2009), and sepsis (Osterud and Bjorklid, 2001; Aras et al., 2004; Wang et al., 2009). Signaling through PARs plays a critical role for this reciprocal process. TF:FVIIa complex has been implicated in the induction of inflammation in the aforementioned clinical models. In an endotoxemic animal model, both TF deficiency and combined inhibition of thrombin and deficiency in PAR2 reduced inflammation (Pawlinski et al., 2004). Further studies in animal models of sepsis demonstrated that extrinsic coagulation cascade inhibition with a varying range of anticoagulants [natural anticoagulants, Tissue Factor Pathway Inhibitor (TFPI), Protein C, and Antithrombin III] attenuated the persisting inflammation (Taylor et al., 1987, 1998a,b; Ramachandran and Hollenberg, 2008). Moreover, it has been recently shown that thrombin is able to generate biologically active C5a from C5 in the absence of C3, indicating a significant role in the reignition of inflammation (Huber-Lang et al., 2006; Krisinger et al., 2012). However, the physiological contribution of this pathway has to be further investigated. This data establish the reciprocal and close relationship between the two systems.

### TF production by neutrophils: a controversial issue

The expression of TF by neutrophils was initially reported almost 40 years ago (Lerner et al., 1971), although those studies did not include solid proof on their ability to produce TF protein. However, it is less than 10 years since evidence on the ability of neutrophils to produce functional TF arose. A turning point in the investigation of TF expression by blood cells was the observation that the promoter of TF gene is strictly regulated by methylation. TF promoter exists in its unmethylated form in both monocytes and neutrophils, allowing stimuli-driven TF mRNA transcription.

TF production by neutrophils was opposed by other reports (Osterud, 2004, 2012). It was reported that isolated neutrophils failed to produce TF protein when stimulated with LPS alone or in conjunction with phorbol myristate acetate (PMA) or TNF-α (Osterud et al., 2000). TF activity in culture supernatants was attributed to platelet activation by contaminating monocytes (Osterud et al., 2000). A study in an *in vivo* murine sepsis model demonstrated that the cluster of TF positive cells infiltrating the spleen were granulocytes. However, even though these cells expressed TF protein, they did not possess TF mRNA (de Waard et al., 2006). The authors attributed this finding to the uptake by neutrophils of TF produced by other cell types in the form of MPs. This was supported by a study suggesting that neutrophils acquire monocyte-derived TF rather than synthesize it by themselves (Egorina et al., 2008). Using a blood reconstitution model and cells transfected with si-RNA for TF, the authors were able to demonstrate that only monocytes contributed in LPS-induced TF expression. Interestingly, this TF was not localized on neutrophil membrane but intracellularly. Even though there is clear evidence for the acquisition of TF by granulocytes in this article, the authors used a limited number of mediators and conditions for neutrophil activation.

Even though these findings indicate a mechanism for TF transfer from monocytes to neutrophils, they do not exclude the possibility for TF production by neutrophils under *in vivo* inflammatory conditions.

On the opposite side of the debate, it has been found that neutrophils produce functional TF protein after stimulation with P-selecting or N-formyl-methionyl-leucyl-phenylalanine (fMLP) but not PMA. Interestingly, cells expressed TF intracellularly and after stimulation with fMLP the protein was translocated to cell membrane in a small percentage of cells (Maugeri et al., 2006). At the same time, the ability of neutrophils to produce TF was reported in an *ex vivo* human model of APS (Ritis et al., 2006). It was shown that IgG immunoglobulin from patients with APS triggers the activation of complement and subsequently generation of C5a. The produced anaphylatoxin was able to induce *TF* gene transcription and production of active TF by human neutrophils. These findings were verified by another study in a murine *in vivo* APS model. It was demonstrated that TF responsible for fetal miscarriages was derived from myeloid cells and particularly neutrophils in a C5a-dependent manner (Redecha et al., 2007). Blocking of TF attenuated trophoblast damage and reduced miscarriages. The same group in a following study in the same murine model demonstrated that TF:FVIIa complex exhibits signaling through PAR2 receptor on activated neutrophils causing trophoblast damage and fetal death via reactive oxygen species release (Redecha et al., 2008). Mice with deletion of the cytoplasmic domain of TF, responsible for interaction with FVIIa and consequently PAR2 receptor, or PAR2 knockout exhibited lower neutrophil activation levels and normal pregnancies. Moreover, another study demonstrated the expression of TF by neutrophils isolated from the bronchoalveolar fluid (BALF) from patients with Acute Respiratory Distress Syndrome (ARDS) (Kambas et al., 2008). The ability of BALF from such patients to induce TF expression was attributed to the synergistic effect of C5a and TNF-α. C5a-dependent TF production by neutrophils was also observed in an extracorporeal circulation model (Kourtzelis et al., 2010). It was demonstrated that serum from End Stage Renal Disease (ESRD) patients induces functional TF production in both neutrophils and monocytes from healthy individuals in a C5a-dependent manner. Of interest, in the same study, granulocyte colony stimulating factor (G-CSF) levels were significantly correlated with TF expression. The significance of neutrophil priming in their activation by cytokines has been recently described (Yousefi et al., 2009; Hattar et al., 2010). Moreover, *in vitro* data supported the production of TF by neutrophils stimulated with leptin. Inhibition studies indicated that TF induction was partially mediated by TNF-α and JAK2/PI3K (Rafail et al., 2008).

Even though these data provide significant evidence for the ability of neutrophils to produce TF, they do not answer the question on how neutrophils can externalize TF in a functional manner. New breakthroughs in neutrophil biology and particularly the description of NETs reveal possible solutions to unwinding Ariadne's thread regarding this debatable issue (Table 1).

**Table 1:** **Neutrophils, TF, and thrombosis—the historical overview**.

**Year**	**1971**	**2000**	**2006**	**2007**	**2008**	**2010**	**2012**
For	“First evidence on neutrophils producing TF”—Lerner et al. *Proc. Soc. Exp. Biol. Med.*		“Neutrophils produce TF when stimulated with fMLP or P-selectin”—Maugeri et al. *J. Thromb. Haemost.*	“Neutrophils produce TF in *in vivo* APS murine model in a C5a dependent manner”—Redecha et al. *Blood*	“Neutrophils produce TF in ARDS in a C5a and TNF-α dependent manner”—Kambas et al. *J. Immunol.*	“NETs entrap platelets and act as a scaffold for fibrin formation in baboon DVT”—Fuchs et al. *Proc. Natl. Acad. Sci. U.S.A*.	“Neutrophil depletion attenuates thrombosis in mouse DVT. Key role of NETs in DVT”—von Brühl et al. *J. Clin. Invest.*
			“C5a-dependent TF production by neutrophils in APS”—Ritis et al. *J. Immunol.*	“Endotoxemic mice entrap and activate platelets in NETs”—Clark et al. *Nat. Med.*	“TF produced by neutrophils exert autocrine signaling through TF/FVIIa complex in APS”—Reinhardt et al. *J. Clin. Invest.*	“TF production by neutrophils in ESRD patients”—Kourtzelis et al. *Blood*	“Neutrophil TF is critical in laser induced endothelial injury”—Darbousset et al. *Blood*
							“TF delivery in NETs through autophagy in human sepsis”—Kambas et al. *PLoS ONE*
Against		“Neutrophils cannot produce TF when stimulated with LPS and PMA or TNF-α”—Osterud et al. *Thromb. Haemost.*	“In murine sepsis, granulocytes express TF protein but no TF mRNA transcripts”—de Waard et al. *Thromb. Haemost.*		“Neutrophils do not produce TF but acquire it from monocytes when stimulated with LPS and TNF-α or PMA”—Egorina et al. *Blood*		

### A critical role of neutrophils in thrombosis

Despite the debate whether neutrophils are able to produce TF or not, there are several articles demonstrating their active role in *in vivo* experimental thrombosis and inflammation-driven thrombotic diseases. In animal models of acute lung injury caused by differential etiology, including acid aspiration (Folkesson et al., 1995), ischemia/reperfusion (Eppinger et al., 1997), and transfusion related acute lung injury (TRALI) (Looney et al., 2006), neutrophil depletion before the initiation of inflammation attenuates lung injury. Furthermore, in a model of diet-induced atherosclerosis, circulating neutrophils were required for plaque formation in atherosclerotic lesions (Zernecke et al., 2008). An additional contribution of neutrophils in the activation of extrinsic coagulation cascade is the degradation of TFPI *via* elastase release (Massberg et al., 2010). TFPI is the main inhibitor of TF. Additionally, protein disulfide isomerise, a key protein for the activation of cytoplasmic TF (Reinhardt et al., 2008) was found to be expressed in neutrophils (von Brühl et al., 2012). Thus, neutrophils play an important role in the activation of extrinsic coagulation system, either by regulating its breaks or by activating its initiator.

Recently, Darbousset et al. (2012) clearly demonstrated that neutrophil binding to the injured endothelium was the initial step in the continuum of events that resulted in thrombus formation in a model of laser-induced endothelial injury. The critical role of neutrophils was reinforced by the observation that these cells were the main source of TF, which was required for thrombus formation. Additionally, in the same model, factor XII deficiency did not attenuate thrombus generation. Another recent study in a mouse model of deep vein thrombosis (DVT) provided evidence for the indispensable role of neutrophils in venous thrombosis, as shown by neutrophil depletion (von Brühl et al., 2012). Neutrophils were shown to promote thrombogenesis by binding and activating factor XII, which is in contrast to the study by Darbousset et al. using conditional mutants and bone marrow chimeras, the authors also demonstrated that TF derived from myeloid cells and not endothelial cells was responsible for the activation of coagulation system. However, monocyte-derived TF was not sufficient for the formation of thrombus. These well-organized studies provide convincing evidence for the significance of neutrophils in thrombosis and mark the restoration of neutrophils in the forefront of the investigation of thrombosis.

## Nets and thrombosis

Neutrophils, as a critical part of innate immunity, have evolved mainly around their ability to fight bacterial infections. Novel insight on neutrophil biology demonstrated a new mechanism of neutrophils to defend against pathogens through the release of NETs. NETs are chromatin filaments that form a network of DNA, histones and several cytoplasmic and granule proteins with antibacterial or immune-modulating role (Jaillon et al., 2007; Sangaletti et al., 2012). NETs are released as a last measure of defence from neutrophils (Brinkmann and Zychlinsky, 2007).

Recent studies demonstrated the critical implication of NETs in animal thrombotic models. Using a murine model of endotoxemia and an *in vitro* model of blood flow, Clark et al. (2007) reported that the formation of NETs in the vasculature resulted in the entrapment of platelets. Subsequent platelet activation induced endothelial injury leading in the impairment of blood flow. A few years later, the contribution of NETs in thrombus formation was shown in a baboon DVT model (Fuchs et al., 2010). This study demonstrated that NETs entrapped both platelets and erythrocytes, while NETs served as three dimensional scaffolds for fibrin deposition and the subsequent stabilization of thrombus. Moreover, histones were identified as the culprit for platelet activation. Another study demonstrated that neutrophils contribute in tissue injury through NET release in a TRALI mouse model (Caudrillier et al., 2012). More specifically, activated platelets were able to stimulate neutrophils for NET release which increased endothelial permeability. Moreover, inhibition of platelet activation reduced NET release and tissue injury. Pretreatment with either histone blocking or DNase I reduced endothelial damage in TRALI. In a murine DVT model, neutrophils were demonstrated as a major factor of thrombosis, as shown by the effect of neutrophil depletion. Neutrophils were found to contribute to DVT through NET release since treatment with DNase I suppressed DVT growth (von Brühl et al., 2012). Citrullinated H3 histone interaction with von Willebrand factor was proposed as a possible mechanism for the formation of erythrocyte rich thrombus (red thrombus). NET formation was also observed by neutrophils in the context of chronic myelogenous leukemia and in solid tumor models (Demers et al., 2012). The authors correlated NET release with cancer related thrombosis, through presence of citrullinated H3 histone and high plasma DNA concentrations. Finally, as already mentioned, NET release was associated with venous thrombosis through factor XII activation in a murine DVT model induced by partial vessel occlusion.

## Extracellular TF delivery through nets

The contribution of neutrophils in thrombosis was overshadowed by the debate on their ability to produce functional TF. Additionally, the intracellular localization of TF in neutrophils raised concerns regarding its ability to activate coagulation system, since only minimal TF amounts were detected on cell membrane.

An explanation for the extracellular delivery of neutrophil-borne TF was the identification of TF on NETs. The first description of TF expression in NETs was by the study of von Brühl et al. (2012). The authors proposed that activation of factor XII rather than TF on NETs was not essential for thrombus formation even though they did not investigate the role of TF present on NETs. Recently, we demonstrated that neutrophils from patients with sepsis release large amounts of TF in the form of NETs (Kambas et al., 2012). NET-borne TF was able to generate thrombin, which subsequently resulted in platelet activation. Microparticle depletion from sepsis serum suggested the *de novo* TF production and ensured that neutrophils did not acquire TF from TF-bearing MPs of unknown origin. The observed inclusion of TF in autophagosomes prior to its extracellular delivery in NETs suggested the involvement of autophagy in this process. This autophagy-dependent pathway was also shown for high-mobility group protein B1 (HMGB-1), proposing a role for autophagy as a secretory mechanism for the externalization of membrane bound or cytosolic proteins to NETs. Using *in vitro* stimulation studies, it was shown that neutrophil priming with pro-inflammatory cytokines is required for TF mRNA translation after bacterial phagocytosis. These findings further demonstrate the critical role of inflammatory mediators in the post-transcriptional regulation of TF. Even though there are speculations that *in vivo* NET-bound TF could be trapped MPs of different origin (neutrophil, platelet, monocyte, etc.), it does not alter the crucial role of NETs in the interplay between inflammation and thrombosis.

**Figure 1:**
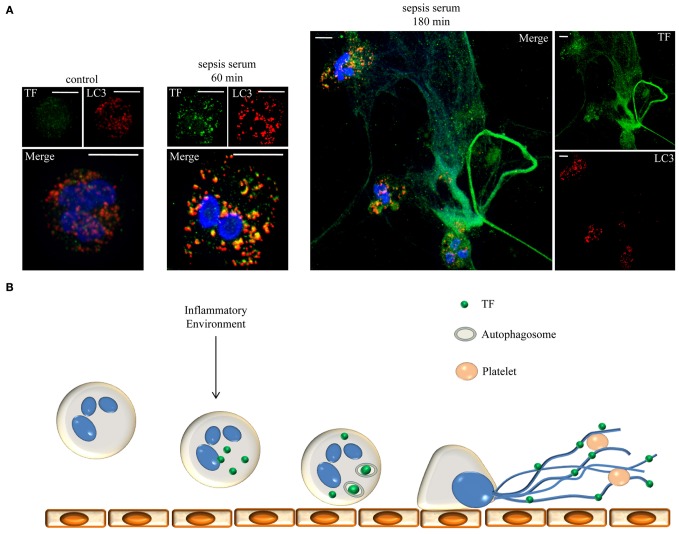
**Neutrophils release NETs decorated with TF under sepsis conditions. (A)** Neutrophils were incubated for 60 or 180 min with serum from patients with sepsis to release NETs. TF (green staining) is localized in autophagosomes (LC3B: red staining) and in NETs, respectively. DNA stained with DAPI (blue). Z-stack analysis. Scale bars represent 5 μM. Original magnification 1000×. **(B)** Schematic representation of the proposed mechanism. Inflammatory conditions prime and stimulate neutrophils to produce TF which is engulfed in autophagosomes and translocated on NETs. TF coated NETs can further entrap circulating platelets to form thrombus and trigger cell signaling through PARs.

Based on the above findings, NETs act as mechanism for the localized extracellular expression of intracellular epitopes and anti-microbial proteins. These networks function as a scaffold for thrombus formation by exposing active TF to the other serine proteases of the extrinsic coagulation cascade. Moreover, entrapment and activation of circulating platelets further contributes in the obstruction of blood flow, while entrapped platelets prevent the degradation of this scaffold by DNase. This maelstrom of events, from neutrophil attachment to endothelium to NET release and the subsequent thrombi formation in the microvasculature could be critical in the pathophysiology of sepsis (Figure 1). Autophagy could function as a selective transport mechanism for the delivery of proteins to NETs. Moreover, an additional role for autophagy in the regulation of protein levels through degradation or even post-translation protein modification is implied.

## Conclusion

Despite the well-established correlation between inflammation and thrombosis in clinical practice (Aras et al., 2004; Miehsler et al., 2004; Tomasson et al., 2009; Zöller et al., 2012), the role of neutrophils in thrombogenicity has only recently emerged. Growing evidence support the critical involvement of NET formation in this process. Neutrophil recruitment and activation at the site of endothelial damage is considered as the initial event in thrombus formation. The local intralluminal exposure of high levels of thrombogenic TF in NETs could be essential for the initiation and propagation of both venous and arterial thrombosis. Additionally, neutrophil-driven activation of the extrinsic coagulation cascade could exert through PAR signaling a significant contribution in non-thrombotic processes, including inflammation, cancer biology, or fibrosis.

### Conflict of interest statement

The authors declare that the research was conducted in the absence of any commercial or financial relationships that could be construed as a potential conflict of interest.
